# Molecular and functional expression of anion exchangers in cultured normal human nasal epithelial cells

**DOI:** 10.1111/j.1748-1716.2007.01731.x

**Published:** 2007-10

**Authors:** J-H Shin, E J Son, H S Lee, S J Kim, K Kim, J Y Choi, M G Lee, J-H Yoon

**Affiliations:** 1 The Airway Mucus Institute, Yonsei University College of Medicine Seoul, Korea; 2 Brain Korea 21 Project for Medical Science, Yonsei University College of Medicine Seoul, Korea; 3 Department of Otorhinolaryngology, National Medical Center Seoul, Korea; 4 Department of Otorhinolaryngology, Yonsei University College of Medicine Seoul, Korea; 5 Biomolecule Secretion Research Center, Yonsei University College of Medicine Seoul, Korea; 6 Department of Pharmacology, Yonsei University College of Medicine Seoul, Korea

**Keywords:** anion exchangers, nasal epithelium, cell differentiation

## Abstract

**Aims:**

Anions have an important role in the regulation of airway surface liquid (ASL) volume, viscosity and pH. However, functional localization and regulation of anion exchangers (AEs) have not been clearly described. The aim of this study was to investigate the regulation of AE mRNA expression level in accordance with mucociliary differentiation and the functional expression of AEs cultured normal human nasal epithelial (NHNE) cells.

**Methods:**

Nasal mucosal specimens from three patients are obtained and serially cultured cells are subjected to morphological examinations, RT-PCR, Western blot analysis and immunocytochemistry. AE activity is assessed by pHi measurements.

**Results:**

Expression of ciliated cells on the apical membrane and expression of MUC5AC, a marker of mucous differentiation, increased with time. AE2 and SLC26A4 mRNA expression decreased as mucociliary differentiation progressed, and AE4, SLC26A7 and SLC26A8 mRNA expression increased on the 14th and 28th day after confluence. Accordingly, AE4 protein expression also progressively increased. AE activity in 100 mm K^+^ buffer solutions was nearly twofold higher than that in 5 mm K^+^ buffer solutions. Moreover, only luminal AE activity increased about fourfold over the control in the presence of 5 μm forskolin. In the presence of 100 μm adenosine-5′-triphosphate (ATP) which evokes intracellular calcium signalling through activation of purinergic receptors, only luminal AE activity was again significantly increased. On the other hand, 500 μm 4,4′-diisothiocyanostilbene-2,2′-disulfonic acid (DIDS), an inhibitor of most SLC4 and SLC26AE isoforms, nearly abolished AE activity in both luminal and basolateral membranes. We found that AE activity was affected by intracellular cAMP and calcium signalling in the luminal membrane and was DIDS-sensitive in both membranes of cultured NHNE cells.

**Conclusion:**

Our findings through molecular and functional studies using cultured NHNE cells suggest that AEs may have an important role in the regulation of ASL.

Airway epithelial cells regulate fluid and ion absorption and secretion to maintain pH and volume of airway surface liquid (ASL) (Smith & Welsh 1992, Van Scott *et al.* 1995, Coakley *et al.* 2003, Hunt 2006). The nasal epithelium, as part of the airway system, is known to actively secrete mucin and determine the electrolyte composition of nasal secretions. Disruption of these processes can lead to altered composition of nasal secretions and impairment of mucociliary clearance, common features of various nasal and paranasal diseases (Tos & Mogensen 1984). However, little is known about the mechanisms of fluid and electrolyte secretion and absorption in the human nasal epithelium.

The anions HCO_3_^−^ and Cl^−^ are major constituents of cellular secretion and also affect ASL pH (Coakley & Boucher 2001). Various anion transporters, such as the anion exchangers (AEs), cystic fibrosis transmembrane conductance regulator (CFTR), sodium bicarbonate cotransporter (NBC) and calcium activated chloride channel (CaCC) are expressed in the human airway (Jacquot *et al.* 1993, Inglis *et al.* 2002, Mall *et al.* 2003, Paradiso *et al.* 2003). AEs translocate monovalent anions such as Cl^−^ and HCO_3_^−^ across the plasma membranes. Recent studies have identified two unrelated multigene families of AEs: the SLC4 and SLC26 transporters (Mount & Romero 2004, Alper 2006). The SLC4 transporter family of 10 genes includes three types of HCO_3_^−^ transporters: AE (exchanges Cl^−^ and HCO_3_^−^), NBC (transports Na^+^ and HCO_3_^−^) and Na^+^-driven Cl^−^–HCO_3_^−^ exchangers (NDCBE). Four genes of this SLC4 family, SLC4A1, -A2, -A3 and SLC4A9, were reported as Cl^−^–HCO_3_^−^ exchangers and these isoforms were named AE1, AE2, AE3 (bAE3 and cAE3) and AE4, respectively (Romero 2005). The SLC26 family consists of 10 genes (SLC26A1 to -A9 and -A11) encoding AEs that transport various anions including SO_4_^2−^, Cl^−^, I^−^, OH^−^ and HCO_3_^−^ with variable specificity (Mount & Romero 2004).

Among various isoforms, only AE2, bAE3 and SLC26A9 have been shown by RT-PCR to be expressed in human tracheobronchial tree and lung cancer cell lines (Al-Bazzaz *et al.* 2001, Lohi *et al.* 2002). Moreover, the possible effect of mucociliary differentiation on the expression of AE has not been reported in cultured airway epithelia. Recently, functional characterization of AEs has been investigated with pharmacological methods in various human cells and tissue including distal colon (Taylor *et al.* 2001) and pancreatic duct cell lines (Cheng *et al.* 1998). However, there are only a few reports on the function of AEs in the human airway, including the nasal epithelia (Loffing *et al.* 2000, Inglis *et al.* 2002, Mall *et al.* 2003). Therefore, the aim of this study was to investigate the regulation of mRNA expression of AEs in accordance with mucociliary differentiation, and the functional expression of AEs in both luminal and basolateral membranes in cultured normal human nasal epithelial (NHNE) cells. To this purpose, we first induced mucociliary differentiation of cultured NHNE cells and then confirmed differentiation with histological and molecular characterization. Next, we identified different types of AE isoforms present and any changes with respect to mucociliary differentiation in cultured NHNE cells. Finally, we examined the functional activity of AEs in the luminal and basolateral membranes of polarized monolayers of cultured NHNE cells.

## Materials and methods

### Cell culture

Human nasal tissues were obtained from the inferior turbinate mucosa of three patients during surgery for septal deviation or maxillary sinus cancer. In patients with maxillary sinus cancer, specimens were gathered from the macroscopically normal areas adjacent to the tumour. This procedure was approved by the Institutional Review Board of Yonsei University College of Medicine. Passage-2 NHNE cells (1 × 10^5^ cells) were seeded in 0.5 mL of culture medium onto 24.5 mm, 0.45 μm -pore Transwell-clear (Costar, Cambridge, MA, USA) culture inserts. The cells were cultured using a 1 : 1 mixture of bronchial epithelial growth medium (BEGM) and Dulbecco's modified Eagle's medium (DMEM) containing the supplements listed in Table 1 (Yoon *et al.* 1997). Cultures were maintained at 37 °C in an atmosphere of 5% CO_2_ in air.

**Table 1:** Supplements of normal human nasal epithelial cell culture media

Supplement	Concentration
Hydrocortisone 21-hemisuccinate	0.5 μg mL^−1^
Insulin	5 μg mL^−1^
Transferrin	10 μg mL^−1^
Epinephrine hydrochloride	0.5 μg mL^−1^
3,3′5-triido-l-thyronine	6.5 ng mL^−1^
Gentamicin sulfate	50 μg mL^−1^
Amphotericin B	50 μg mL^−1^
Epidermal growth factor	25 ng mL^−1^
All-trans RA	10^−7^mol L^−1^
Bovine serum albumin	1.5 μg mL^−1^
Bovine pituitary extract	1% vol vol^−1^

The cultures were grown submerged for the first 9 days, during which the culture medium was changed daily. An air-liquid interface (ALI) was created on the 9th day by removing the apical medium and feeding the cultures only from the basal compartment. The culture medium was changed daily after the ALI was generated (Yoon *et al.* 2000). In order to determine time duration effects, RNA was collected on the day of confluence and on the 7th, 14th and 28th days after confluence.

### Morphologic examination

To examine cell differentiation, cultured cells on the semi-permeable membrane were fixed in 10% buffered neutral formalin, embedded in paraffin, and then sectioned. The slides were stained with haematoxylin and eosin and observed with a light microscope (Olympus Light Microscope, Vanox-S type, Japan) on the day of confluence and on the 7th, 14th and 28th day after confluence.

### Periodic acid-Schiff staining

Serial sections were collected on coated slides. The slide sections were treated with 3% glacial acetic acid for 3 min, incubated with 0.5% periodic acid (Fisher Scientific, Pittsburgh, PA, USA) solution for 5 min, and rinsed with phosphate-buffered saline (PBS). The sections were then reacted with Schiff's solution (Sigma Chemical Company, St. Louis, MO, USA) for 5 min, washed in tap water, and counterstained with Harris haematoxylin.

### RT-PCR

Gene-specific PCR primer sets for human AE2, bAE3, AE4 and SLC26A3-A11 (except SLC26A5 and SLC26A10) were designed to detect isoform-specific mRNA in human airways. MUC5AC and cornifin-α primer sets were used to confirm the differentiation of NHNE cells according to culture period. The sequence information of some isoforms was retrieved from GenBank. Oligonucleotide amplimers for β_2_ microglobulin, which generated a 266 bp PCR fragment, were used as the control gene for RT-PCR (Table 2). Total RNAs were collected from cultured NHNE cells using Trizol solution (Gibco BRL, Rockville, MA, USA), and PCR was performed. We used comparative kinetic analysis to compare mRNA levels for each gene and for each set of culture conditions. The linear range for each PCR was established by plotting the intensity of the signal vs. the PCR cycle number. The linear range for each AE isoform was found to be between 25 and 30 cycles. RT-PCR products were separated by electrophoresis on a 2% agarose gel containing 50 ng mL^−1^ ethidium bromide. Bands of the expected sizes were visualized under ultraviolet light and photographed with Polaroid Type 55 film. Negative controls were performed by omitting reverse transcriptase from the RT reactions to verify that the amplified products were from the mRNA and did not originate from genomic DNA contamination. No PCR products were observed in the absence of reverse transcriptase. Specific amplification of all target genes was confirmed by sequencing (ABI PRISM 3100; Applied Biosystems, Foster City, CA, USA) the PCR fragments (dsDNA Cycle Sequencing System, Gibco BRL, Rockville, MA, USA).

**Table 2:** PCR primer sequences specific to the target genes and annealing temperatures

Primer	Sequence	Annealing temperatures (°C)	Size (bp)	Cycle
AE2	Sense: 5′-GAAGATTCCTGAGAATGCCT-3′	**55.5**	**181**	**30**
	Antisense: 5′-GTCCATGTTGGCAGTAGTCG-3′			
bAE3	Sense: 5′-ATCTGAGGCAGAACCTGTGG-3′	**60**	**418**	**28**
	Antisense: 5′-TTTCACTAAGTGTCGCCGC-3′			
AE4	Sense: 5′-AGCGCTTGGACTGCCTTGGTATGT-3′	**57**	**431**	**30**
	Antisense: 5′-AGGGGGAAGATGATGGCTGCAGGGGTAGAC-3′			
SLC26A3	Sense: 5′-TGCCACAGCCAACAGAAAAATCAAA-3′	**58**	**330**	**30**
	Antisense: 5′-GGGGGAATGTCGACCAGCAGAG-3′			
SLC26A4	Sense: 5′-GTTTACTAGCTGGCCTTATATTTGGACTGT-3′	**55**	**484**	**30**
	Antisense: 5′-AGGCTATGGATTGGCACTTTGGGAACG-3′			
SLC26A6	Sense: 5′-TAGGGGAGGTTGGGCCAGGGATGC-3′	**60**	**456**	**28**
	Antisense: 5′-TGCCGGGAAGTGCCAAACAGGAAGAAGTAGAT-3′			
SLC26A7	Sense: 5′-CACTGTGTCTGGGATAATGTTGG-3′	**65**	**353**	**30**
	Antisense: 5′-CCAGTTGCAGCACAAACATG-3′			
SLC26A8	Sense: 5′-CCAAGACCCAGACCGAGATG-3′	**58**	**150**	**30**
	Antisense: 5′-GAGTCTGAGACTGGGTGGAAGC-3′			
SLC26A9	Sense: 5′-TCCAGGTCTTCAACAATGCCAC-3′	**58**	**400**	**30**
	Antisense: 5′-CGAGTCTTGTGCATGTAGCGAG-3′			
SLC26A11	Sense: 5′-ATC CCG CCC TTC TCA GTG AC-3′	**65**	**329**	**28**
	Antisense: 5′-TAGTCCAGAGACAGCAGCACCAG-3′			
MUC5AC	Sense: 5′-TCCGGCTCCATCTTCTCC-3′	**60**	**680**	**33**
	Antisense: 5′-ACTTGGGCACTGGTGCTG-3′			
cornifin-α	Sense: 5′-CATTCTGTCTCCCCCAAAAA-3′	**60**	**172**	**30**
	Antisense: 5′-ATGGGGGTATAAGGGAGCTG-3′			
β_2_ microglobulin	Sense: 5′-CTCGCGCTACTCTCTCTTTCTGG-3′	**55**	**266**	**23**
	Antisense: 5′-GCTTACATCTCTCCATCCCACTTAA-3′			

### Western blot analysis

Cell lysates were collected on the day of confluence and on the 7th, 14th and 28th days after confluence to investigate the presence of select proteins. NHNE cells were lysated and boiled for 5 min in a sample buffer, separated by SDS–PAGE on 6% acrylamide minigels and blotted onto nitrocellulose membranes. After incubation in a blocking buffer, the membranes were treated with diluted 1 : 1000 anti-AE4 antibodies (Alpha Diagnostic International, San Antonio, TX, USA) and then with horseradish peroxidase-conjugated anti-rabbit IgG (BioRad, Richmond, CA, USA) as the secondary antibody. The signal was detected by means of enhanced chemiluminescence (ECL plus system; Amersham, Aylesbury, UK).

### Fluorescent immunocytochemistry

For fluorescent immunohistochemistry, cells were washed three times with PBS and fixed in 3% paraformaldehyde solution [3% (wt vol^−1^) paraformaldehyde, 0.1 mm CaCl_2_ and 0.1 mm MgCl_2_, pH 7.4, in PBS] for 10 min. The cells were then washed three times with PBS, permeabilized in 0.2% Triton® X-100/PBS (Merck, Darmstadt, Germany) for 5 min, washed three more times with PBS, blocked with 10% normal goat serum (Jackson ImmunoResearch, West Grove, PA, USA) for 1 h, and then washed with PBS again. AE4 proteins were detected using an AE4 polyclonal antibody (1 : 100, Alpha Diagnostic International, San Antonio, TX, USA) incubated for 24 h at 4 °C. The cells were then washed with PBS, and the above procedure was repeated with an appropriate fluorescein isothiocyanate (FITC)-conjugated secondary antibody (1 : 100, Jackson ImmunoResearch, West Grove, PA, USA). Cover slips were subsequently mounted on the slides with Vectashield Mounting Medium (Vector Laboratories, Burlingame, CA, USA). Slides were examined using a Zeiss LSM 510 confocal microscope (Carl Zeiss, Thornwood, NY, USA). Negative control experiments were performed by overnight incubation with an anti-rabbit IgG (1 : 100, Jackson ImmunoResearch) instead of primary antibody.

### Measurement of pHi

On the day of confluence, NHNE cells were washed twice with a *N*-2-hydroxyethylpiperazine-*N*–2- (HEPES) solution and incubated in the same solution containing 1 μm 2′, 7′-bis(2-carboxyethyl)-5(6)-carboxyfluorescein (BCECF/AM). BCECF/AM itself is not fluorescent, but it is converted to fluorescent BCECF via the action of intracellular esterase. Cells were loaded with BCECF by incubation for 15–20 min at room temperature. The cells were then mounted in a miniature Ussing chamber (AKI Institute, University of Copenhagen, Denmark) attached to the stage of an inverted microscope. The Ussing chamber consisted of top (mucosal) and bottom (serosal) half-chambers (volume = 250 μL), each made from light-absorbing polyacetal. The Transwell wafer containing the polarized epithelial monolayer was mounted between the two-half chambers with the mucosal surface facing upwards. Effective sealing was achieved using rubber *O*-rings embedded in the grooves of the two-half chambers, which were screwed tightly together. A glass cover slip was affixed to the bottom of the serosal chamber with dental sticking wax (model Deiberit-502; Ludwig Bohme, Bad Sachsa/Harz, Germany). The mucosal chamber was open to the atmosphere. Both half chambers had inlet and outlet ports to allow solution flow. The serosal and mucosal perfusates were heated to 37 °C and delivered to the chamber by gravity flow (rate = 3–5 mL min^−1^). The BCECF/AM fluorescence ratio was recorded (Photon Technology International Delta Ram, NJ, USA) from an area in the centre of the epithelium at excitation wavelengths of 440 and 490 nm and fluorescence emission intensity was recorded at 520 nm. The 490/440 ratios were calibrated intracellularly by perfusing the cells with solutions containing 145 mm KCl, 10 mm HEPES and 5 μm nigericin with the pH adjusted to 6.2–7.6. [Cl^−^]_i_/[HCO_3_^−^]_o_ exchange activities were estimated from initial rate of pHi increase as a result of [Cl^−^]_o_ removal. Initial rates of pHi changes were obtained from the first derivative of the traces using a single exponential fit (Lee *et al.* 1999b).

### Solutions and chemicals

The HCO_3_^−^ buffered Cl^−^ free solution contained (mm) 115 Na-gluconate, 5 K-gluconate, 1 MgSO_4_, 1 Ca cyclamate, 10 d-glucose, 5 HEPES and 25 NaHCO_3_ (pH 7.4 with NaOH). The HCO_3_^−^ -buffered high K^+^ (100 mm K^+^) content solution contained (mm) 25 NaCl, 100 KCl, 1 MgCl_2_, 1 CaCl_2_, 10 Glucose, 5 HEPES and 25 NaHCO_3_. All solutions were adjusted to pH 7.4 with NaOH and HCO_3_^−^. All HCO_3_^−^ -buffered solutions were continuously gassed with 95% O_2_ and 5% CO_2_ to maintain solution pH. The osmolarity of all solutions was adjusted to 310 mOsm with the major salt prior to use. BCECF-AM was purchased from Molecular Probes (Eugene, OR, USA), Forskolin was purchased from Calbiochem and 1,2-bis(2-aminophenoxy)ethane-N,N,N,N’-tetraacetic acid acetoxymethyl ester (BAPTA-AM), diphenylamine-2-carboxylic acid (DPC), adenosine-5′-triphosphate (ATP) and 4,4′-diisothiocyanostilbene-2,2′-disulfonic acid (DIDS) were purchased from Sigma.

### Statistical analyses

Data are presented as original recordings and as mean values ± SD from *n* observations. Differences were tested with anova and a *P*-value < 0.05 was considered statistically significant.

## Results

### Induction of mucociliary differentiation of cultured NHNE cells

**Figure 1:**
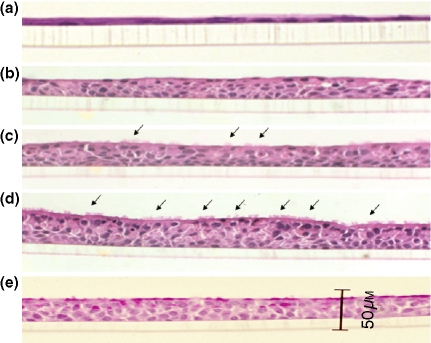
Histological appearance of mucociliary differentiation of NHNE cells over time. At the time of confluence, there was only a monolayer of cells (a). On the 7th day after confluence, the cells grew to form several layers (b). On the 14th day after confluence, ciliated cells could occasionally be seen (c). On the 28th day after confluence, the amount of ciliated cells was greater and the cells themselves became more cuboidal (d). To see whether these cells were mucous cells, they were stained with PAS solution. Many cells containing mucus could be observed (e). Arrows indicate the ciliated cells.

**Figure 2:**
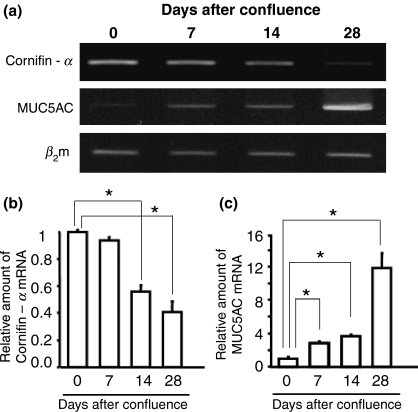
Molecular characterization of mucociliary differentiation of normal human nasal epithelial (NHNE) cells over time. The gene expression of cornifin-α, a marker of squamous cell differentiation, decreased progressively on the 14th and 28th day after confluence. In contrast, the expression of MUC5AC, a marker of mucous differentiation, increased as time passed by. β_2_-microglobulin expression, used as a control, was not altered (a). The figure shows the mean ± SD of separate experiments performed under each condition (b, c). An asterisk indicates statistical significance (*P* < 0.05).

Cultured NHNE cells were analysed histologically as a function of time on the 0, 7th, 14th and 28th day after confluence. The cells differentiated into mucociliary epithelia with time in the presence of 10^−7^ m retinoic acid. Only a monolayer of cells was present at the time of confluence (Fig. 1a). On the 7th day after confluence, the cells grew to form several layers (Fig. 1b). On the 14th day after confluence, ciliated cells were seen occasionally (Fig. 1c). On the 28th day after confluence, the number of ciliated cells was greater and the cellular shape became more cuboidal (Fig. 1d). Periodic acid-Schiff (PAS) staining was performed to see whether these cells were mucous cells, and many cells containing mucus were observed (Fig. 1e). To confirm such mucociliary differentiation on a molecular level, we performed RT-PCR. The gene expression of cornifin-α, a marker of squamous cell differentiation, decreased progressively on the 14th and 28th day after confluence. In contrast, the expression of MUC5AC, a marker of mucous differentiation, increased as time passed by. The expression of β_2_ microglobulin, used as control, was not altered (Fig. 2a). The experiment was performed three separate times. Semi-quantitation of the three experiments was performed for each target gene (Fig. 2b,c).

### Expression of AE isoforms as a function of mucociliary differentiation in cultured NHNE cells

**Figure 3:**
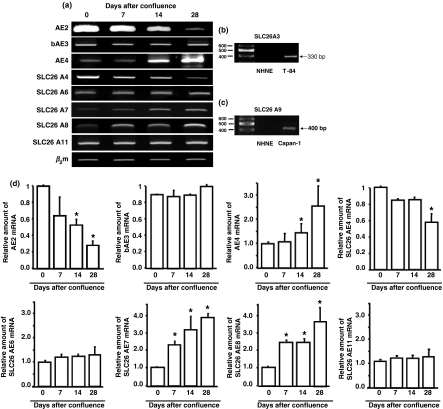
Expression of anion exchanger (AE) isoforms as a function of mucociliary differentiation in normal human nasal epithelial (NHNE) cells. The mRNAs of AE2, bAE3, AE4, SLC26A4, SLC26A6, SLC26A7, SLC26A8 and SLC26A11 were expressed in NHNE cells (a). However, the mRNAs of SLC26A3 and SLC26A9 were not expressed. To confirm that SLC26A3 and SLC26A9 are not expressed in NHNE cells, we used T-84 cells, a human colonic cell line, as a positive control for SLC26A3 (b), and Capan-1 cells, a metastatic human pancreatic cancer cell line, for SLC26A9 (c). Relative abundance of each AE isoform was determined by calculating the ratio of its density to the density of β_2_ microglobulin (d). Values are presented as mean ± SD of three independent samples from three donors.

We examined the presence of 10 AE isoforms. The mRNAs of AE2, bAE3, AE4, SLC26A4, SLC26A6, SLC26A7, SLC26A8 and SLC26A11 were expressed in cultured NHNE cells (Fig. 3a). However, the mRNAs of SLC26A3 and SLC26A9 were not expressed. We used T-84 cells, a human colonic cell line, as a positive control for SLC26A3 (Fig. 3b), and Capan-1 cells, a metastatic human pancreatic cancer cell line, for SLC26A9 (Fig. 3c).

Mucociliary differentiation did not affect the mRNA expression levels of AE3, SLC26A6 and SLC26A11. However, AE2 and SLC26A4 mRNA expression levels decreased, and AE4, SLC26A7 and SLC26A8 mRNA expression levels progressively increased on the 14th and 28th day after confluence. The specimens were obtained from three different donors and the experiments were performed three separate times. Semi-quantitation of the three experiments was performed for each target gene (Fig. 3d).

### Expression of the AE4 protein as a function of mucociliary differentiation of NHNE cells

**Figure 4:**
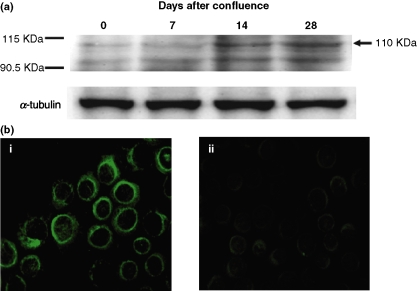
Expression of AE4 protein as a function of differentiation of normal human nasal epithelial (NHNE) cells. Western blot analysis demonstrated that AE4 protein levels increased on the 14th and 28th days after confluence (a). Positive immunofluorescent staining of AE4 was seen along the entire circumference of the cell membranes in fully differentiated NHNE cells (b-i), whereas the negative control generated only faint signals (b-ii). Magnification: ×1000.

To verify the AE expression profile, we performed Western blotting with anti-AE4 antibody at different time points during differentiation and immunofluorescent staining using cytospun slides of the cultured cells at 28 days after confluence. Western blot analysis revealed that AE4 protein expression levels were higher on the 14th and 28th days after confluence (Fig. 4a). This result was consistent with the expression pattern of AE4 mRNA and correlated with mucociliary differentiation. Positive immunofluorescent staining was seen along the entire circumference of the cell membranes in fully differentiated NHNE cells (Fig. 4b-i) whereas a negative control generated only faint signals (Fig. 4b-ii).

### The effect of extracellular K^+^ concentration on AE activity in cultured NHNE cells

**Figure 5:**
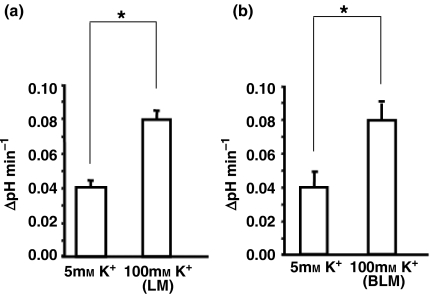
Effect of K^+^ concentration on extracellular Cl^−^ free condition-induced pHi changes. The luminal side was exposed to Cl^−^ free solutions in the presence of 5 or 100 mm K^+^ (a). The basolateral side was exposed to Cl^−^ free solutions in the presence of 5 or 100 mm K^+^ (b).

Changes in Cl^−^/HCO_3_^−^ exchange activity of either luminal or basolateral membranes were estimated from the initial rate of pHi changes caused by removal and addition of Cl^−^ to the perfusing medium while the other membrane was bathed in Cl^−^ free medium. The possibility exists that the rate of HCO_3_^−^ influx during Cl^−^ removal could be underestimated because other electrogenic chloride channels may also function to pump out Cl^−^ ions to lessen the concentration gradient of the ion across the plasma membrane. Thus, the effect of extracellular K^+^ concentration and subsequent membrane depolarization on AE activity in NHNE cells was examined using physiologic buffers that contained either 5 or 100 mm K^+^ (gassed with 5% CO_2_). In the presence of 5 mm K^+^, removal and addition of Cl^−^ to the bathing medium on the luminal membrane resulted in luminal AE activity of 0.040 ± 0.005 ΔpH unit min^−1^, which increased to 0.079 ± 0.007 ΔpH unit min^−1^ in the 100 mm K^+^ solution (Fig. 5a). Likewise, basolateral AE activity was 0.040 ± 0.010 ΔpH unit min^−1^ in the 5 mm K^+^ solution, and 0.083 ± 0.012 ΔpH unit min^−1^ in 100 mm K^+^ solution (Fig. 5b). Experiments after this were performed in high extracellular K^+^ conditions.

### The effect of forskolin on AE functional activity

**Figure 6:**
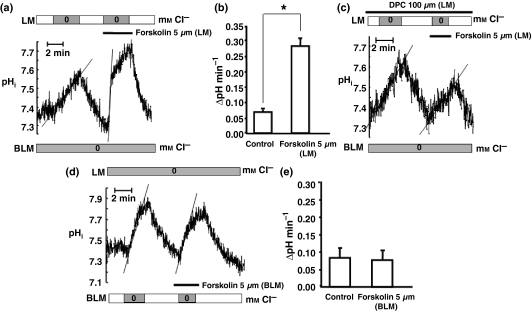
Effect of forskolin on anion exchange functional activity. The effect of forskolin, a cAMP-elevating agent, on anion exchange activity was determined from the extent of pHi changes caused by Cl^−^ removal and addition before and after 5 μm forskolin stimulation (a, b) and diphenylamine-2-carboxylic acid (DPC), a cystic fibrosis transmembrane conductance regulator (CFTR) inhibitor (c), on the luminal membrane. The effect of forskolin on basolateral membrane anion exchange activity was determined from the extent of pHi changes caused by Cl^−^ removal and addition before and after 5 μm forskolin stimulation (d, e). The experiment was performed in high extracellular potassium (100 mm K^+^) conditions. The figure shows the mean ± SD of six separate experiments. An asterisk indicates statistical significance (*P* < 0.05).

In the 100 mm K^+^ solution, the resting pHi of the monolayered NHNE cells was 7.35 ± 0.12 in the presence of HCO_3_^−^. The effect of forskolin, a cAMP-elevating agent, on AE activity was determined from the rate of pHi changes caused by Cl^−^ removal and addition before and after 5 μm forskolin stimulation on each side of the membrane. The rate of luminal AE activity was 0.070 ± 0.009 ΔpH unit min^−1^, which increased to 0.283 ± 0.030 ΔpH unit min^−1^ in the presence of 5 μm forskolin (Fig. 6a,b). Regulation of Cl^−^/HCO_3_^−^ exchange activity by CFTR has been demonstrated in cells either transfected with or naturally expressing CFTR (Lee *et al.* 1999a,b). To examine the possibility of CFTR-dependent anion exchange, the NHNE cell monolayers were pretreated with 100 μm DPC for 1 min at room temperature. Forskolin-stimulated activity of luminal Cl^−^/HCO_3_^−^ exchange was completely inhibited by DPC, which blocked the CFTR-mediated Cl^−^ current (Fig. 6c). On the other hand, basolateral AE activity was 0.087 ± 0.031 ΔpH unit min^−1^, which remained unchanged (0.083 ± 0.026 ΔpH unit min^−1^) after stimulation with 5 μm forskolin (Fig. 6d,e).

### The effect of ATP on AE functional activity

**Figure 7:**
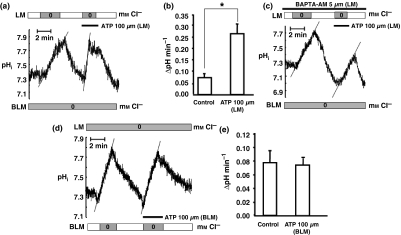
Effect of adenosine-5′-triphosphate (ATP) on anion exchange functional activity. The relationship between anion exchange activity and purinergic receptors was examined by testing normal human nasal epithelial (NHNE) cells with 100 μm ATP (a, b) and 50 μm 1,2-bis(2-aminophenoxy)ethane-N,N,N,N'-tetraacetic acid acetoxymethyl ester (BAPTA-AM), a calcium chelator (c), on the luminal membrane. The effect of adenosine-5′-triphosphate (ATP) on basolateral membrane anion exchange activity was determined from the rate of pHi changes caused by Cl^−^ removal and addition before and after 100 μm ATP stimulation (d, e). The experiment was performed in high extracellular potassium (100 mm K^+^) conditions. The figure shows the mean ± SD of six separate experiments. An asterisk indicates statistical significance (*P* < 0.05).

We previously reported that ATP evoked intracellular calcium signalling in cultured NHNE cells through the activation of luminal and basolateral purinergic receptors (Kim *et al.* 2004). The relationship between AE activity and purinergic receptors was examined by treating the cultured NHNE cells with 100 μm ATP in the 100 mm K^+^ solution. Luminal AE activity increased from 0.071 ± 0.014 ΔpH unit min^−1^ to 0.257 ± 0.040 ΔpH unit min^−1^ following stimulation with 100 μm ATP (Fig. 7a,b). We then preloaded the NHNE cells with 50 μm 1,2-bis(2-aminophenoxy)ethane-N,N,N, N'-tetraacetic acid acetoxymethyl ester BAPTA-AM for 30 min at room temperature before ATP stimulation to investigate Ca^2+^-dependent AE activity. Chelating intracellular calcium with BAPTA-AM completely inhibited the P2R-induced activation of luminal Cl^−^/HCO_3_^−^ exchange (Fig. 7c). However, basolateral AE activity did not increase after basolateral ATP stimulation (Fig. 7d,e).

### The effect of DIDS on AE functional activity

**Figure 8:**
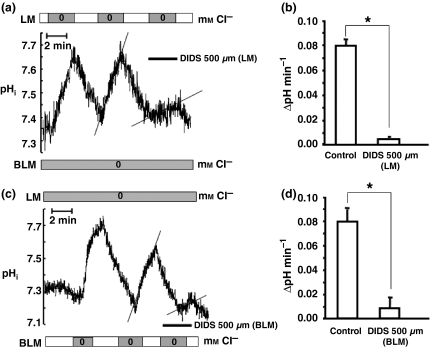
Effect of 4,4′-diisothiocyanostilbene-2,2′-disulfonic acid (DIDS) on anion exchange functional activity. To examine the effect of an anion antagonist on anion exchange activity, we added DIDS to bathing solutions on the luminal (a, b) and basolateral membranes (c, d). The experiment was performed in high extracellular potassium (100 mm K^+^) conditions. The figure shows the mean ± SD of six separate experiments. An asterisk indicates statistical significance (*P* < 0.05).

To examine the effect of an AE inhibitor on NHNE cells, the cells were treated with 500 μm DIDS on either the luminal or basolateral membrane side. In the 100 mm K^+^ solution, the luminal AE activity of 0.080 ± 0.005 ΔpH unit min^−1^ was nearly abolished to 0.005 ± 0.002 ΔpH unit min^−1^ by 500 μm DIDS (Fig. 8a,b). The basolateral AE activity of 0.078 ± 0.011 ΔpH unit min^−1^ dropped to 0.009 ± 0.009 ΔpH unit min^−1^ following the addition of 500 μm DIDS (Fig. 8c,d).

## Discussion

Anion exchangers may play an important role in the regulation of fluid and pHi in cultured NHNE cells. According to other studies, malfunction of some AE isoforms are linked to several diseases in humans. Genetic defects of SCL41 and -A3 are linked to anemia or renal diseases (Alper 2006, Pushkin & Kurtz 2006). Of the SLC26 family, dysfunction of SLC26A2, -A3 and -A4 causes chondrodysplasias, congenital chloride diarrhoea or Pendred syndrome (Mount & Romero 2004). The effect of AE dysfunction is not well known in the human airway, including the nasal mucosa. We conjectured that it may cause cellular eodema and increased secretion, and thus may be associated with diseases, such as allergic rhinitis and vasomotor rhinitis.

We induced mucociliary differentiation of cultured NHNE cells in the presence of retinoic acid and observed the histology of the cultured epithelium at indicated time points. The nasal mucosa is lined by a ciliated pseudostratified columnar epithelium consisting of basal cells, goblet cells and ciliated cells (Petruson *et al.* 1984). On the 28th day after confluence, many ciliated cells and PAS-positive mucus-containing cells were identified. In addition, expression of cornifin-α and MUC5AC correlated to a great degree with the transfiguration of the cultured NHNE cells (Kim *et al.* 2005a). These results indicate that cultured NHNE cells differentiated into mucociliary epithelium in the presence of retinoic acid.

Next, we investigated the expression patterns of AE isoforms as a function of mucociliary differentiation. Among SLC4 family members with a Cl^−^/HCO_3_^−^ exchange function, we only examined AE2, bAE3 and AE4, because AE1 and cAE3 are not expressed in the human airway (Al-Bazzaz *et al.* 2001, Romero 2005). From the SLC26 family, the SLC26A1, -A2, -A5 and -A10 genes were excluded because they are known to have no Cl^−^/HCO_3_^−^ exchange function (Mount & Romero 2004). We found eight types of AE mRNAs expressed in the cultured NHNE cells. AE2 and bAE3 mRNA expression was consistent with previous reports from human bronchial and tracheal epithelium (Dudeja *et al.* 1999, Al-Bazzaz *et al.* 2001). However, mRNAs of SLC26A3, known to be present in mouse tracheal epithelial cells, and SLC26A9, present in human alveolar and bronchial epithelial cells (Wheat *et al.* 2000, Lohi *et al.* 2002), were not expressed in the cultured NHNE cells. This discrepancy may be as a result of the difference in species or the difference in upper and lower airways. Interestingly, expression levels of AE4, SLC26A7 and SLC26A8 mRNAs increased as a function of mucociliary differentiation. In particular, AE4 mRNA and protein expression levels increased significantly on the 14th and 28th day after confluence, correlating with the appearance of ciliated cells. In addition, immunofluorescent staining of cytospun slides was performed on the 28th day after confluence, when the expression of AE4 mRNA was the highest. Positive immunofluorescent staining was seen along the whole cell membrane, which suggests that AE4 is localized in both the luminal and basolateral membranes. Thus, the presence of AE4 in fully differentiated NHNE cells was confirmed. These results suggest that AE4 may function in the regulation of intracellular and ASL pH in fully differentiated mucociliary epithelium *in vivo*.

Little is known about the functional localization of AE in human nasal epithelium. We examined AE functional activity in both luminal and basolateral membranes in a monolayer state. Anion exchange activity was measured as the change (elevation) in pHi because of HCO_3_^−^ ion exchanged into the intracellular compartment driven by the Cl^−^ ion concentration gradient (outward export). When other Cl^−^ exit ion transporters, such as the CaCC or K^+^-Cl^−^ cotransporter are in action, intracellular Cl^−^ decreases rapidly which results in attenuation of the driving force for Cl^−^ influx and HCO_3_^−^ efflux by AE. Cultured NHNE cells are known to express CaCC (Mall *et al.* 2003). The rate of AE activity was nearly twofold higher in 100 mm K^+^ solutions than in 5 mm K^+^ buffer solutions, as expected. In addition, to exclude the possible effect of NBC activity, all experiments were conducted in low Na^+^/high K^+^ conditions. The concentration of Na^+^ in the solutions used (25 mm) was lower than that used to measure NBC activity (140 mm) (Namkung *et al.* 2003), minimizing the effect of NBC activity. Therefore, in the next set of experiments we measured electroneutral Cl^−^/HCO_3_^−^ exchange in low [Na^+^]_o_/high [K^+^]_o_ conditions.

In our experiments, high extracellular K^+^ concentration resulted in depolarization of the cultured NHNE cells. As a result, electrogenic Cl^−^ exit pathways were blocked and most Cl^−^ movement was the result of electroneutral transport. We found that eight isoforms of AE were expressed. SLC4A2, -A3 and -A9 of the SLC4 family have been reported as electroneutral AEs (Alper 2006, Pushkin & Kurtz 2006), and SLC26A4, -A6 and -A7 of the SLC26 family have been reported as electrogenic, while the exact transport mechanisms of SLC26A8 (Lohi *et al.* 2002) and SLC26A11 (Mount & Romero 2004) have not been elucidated. We conclude that our measurements of AE activity using monolayers of cultured cells are not from the function of the electrogenic isoforms.

Anion exchanger is known to be regulated by CFTR in human airway epithelial cell lines (Illek *et al.* 1997). To test possible CFTR-mediated AE functional activity of NHNE cells, we used forskolin, a cAMP-elevating agent. In agreement with other reports (Paradiso *et al.* 2001, Sun *et al.* 2003), AE activity was increased by addition of 5 μm forskolin to the luminal membrane side only. However, such stimulation of AE activity by forskolin was eliminated by DPC, which inhibited the CFTR-mediated Cl^−^ current. These results suggest that luminal AE activity may be affected by cAMP-mediated anion channels such as CFTR, because CFTR is mainly expressed in the luminal membrane (Lee *et al.* 1999a, Loffing *et al.* 2000).

Ca^2+^-activated Cl^−^ channels increase AE activity in the human airway (Paradiso *et al.* 2003, Jeulin *et al.* 2005). ATP, a calcium agonist, increases the [Ca^2+^]_i_ through P2R, which increases HCO_3_^−^ secretion either directly or by way of cAMP-mediated mechanisms (Luo *et al.* 1999, Namkung *et al.* 2003). We measured AE activity in the presence of 100 μm ATP. In our results, only luminal AE activity increased with ATP stimuli, which was inhibited by the calcium chelator BAPTA-AM. In other reports, luminal addition of ATP activates both CaCC and CFTR, and the majority of anion secretion is mediated by the apical P2Y receptor, which leads to CaCC activation (Paradiso *et al.* 2001). Furthermore, we previously reported that the cultured NHNE cells express functionally active P2Y2, P2Y6 and P2Y11 receptors in the luminal membrane (Kim *et al.* 2004). Therefore, the membrane specific functional expression of P2R, CaCC and CFTR may influence luminal AE activity caused by ATP stimuli in NHNE cells.

Anion exchangers activity was affected by both Cl^−^ and HCO_3_^−^ movement. Treatment with DIDS, which inhibits AE function, nearly abolished the AE activity in both luminal and basolateral membranes in cultured NHNE cells. This result is consistent with many other studies where most SLC4 and SLC26 AE isoforms have proven to be DIDS-sensitive (Lohi *et al.* 2002, Xie *et al.* 2002, Mount & Romero 2004, Kim *et al*. 2005b, Romero 2005, Alper 2006, Pushkin & Kurtz 2006).

Limitations of our study include the fact that the experiments for pHi measurements were performed with monolayers of cultured cells on day 0 after confluence. AE measurements on a later stage of the culture would indeed increase the strength of our results. This study focuses on the ion transport mechanisms on the surface epithelial cells. However, using the current methods for AE measurements, it is difficult to observe the signals only from the surface epithelial cells in the multilayered epithelia of later stage cultures. Especially, measurements on the basolateral transporter activity are almost impossible. Perfusate changes in the basolateral side also activate the transporters in the cells of lower layers, because tight junctions are formed along with the surface epithelial cells. Although there are some limitations, we believe our data are still meaningful by presenting the basic characteristics of luminal and basolateral AE of surface epithelial cells that are present throughout the course of differentiation as shown by RT-PCR.

In summary, we demonstrated that cultured human nasal epithelial cells differentiated into mucociliary epithelium and found that the expression of some of AE isoforms correlated with mucociliary differentiation. In addition, we found that AE activity was affected by intracellular cAMP in the luminal membranes and DIDS-sensitive AE activity existed in both luminal and basolateral membranes of cultured NHNE cells. Our findings through molecular and functional studies using cultured NHNE cells suggest that AEs may play an important role in the regulation of intracellular and ASL pH.

## Conflicts of interest

There is no conflict of interest.
